# 3,4,5-Trimethyl-2,4,6-triphenyl-4*H*-thio­pyran

**DOI:** 10.1107/S1600536809005923

**Published:** 2009-02-25

**Authors:** Hossein Rahmani, Hooshang Pirelahi, Seik Weng Ng

**Affiliations:** a Institute of Chemical Industries, Iranian Research Organization for Science and Technology, PO Box 15815-358, Tehran, Iran; b Department of Chemistry, College of Science, University of Tehran, PO Box 13145-143, Tehran, Iran; c Department of Chemistry, University of Malaya, 50603 Kuala Lumpur, Malaysia

## Abstract

The six-membered thio­pyran ring in the title compound, C_26_H_24_S, adopts a boat conformation, with the S atom displaced by 0.478 (2) Å and the 3-methyl­ene C atom by 0.644 (2) Å from the plane of the other four *sp* ^2^-hydridized C atoms. The methyl group on the methyl­ene carbon lies in a pseudo-equatorial position and the phenyl ring in a pseudo-axial position.

## Related literature

For a similar compound, see: Rahmani *et al.* (2009 ▶). For the synthesis, see: Rahmani & Pirelahi (1997 ▶).
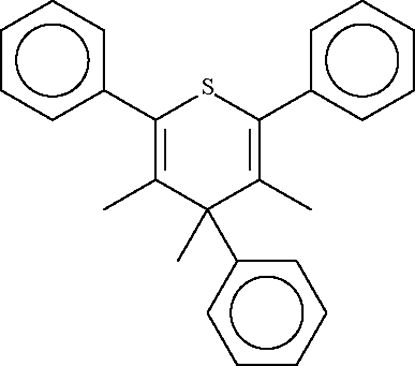


## Experimental

### 

#### Crystal data

C_26_H_24_S
*M* *_r_* = 368.51
Monoclinic, 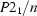

*a* = 8.4525 (1) Å
*b* = 14.4732 (2) Å
*c* = 16.2971 (3) Å
β = 103.156 (1)°
*V* = 1941.37 (5) Å^3^
*Z* = 4
Mo *K*α radiation
μ = 0.17 mm^−1^
*T* = 115 K
0.35 × 0.35 × 0.20 mm

#### Data collection

Bruker SMART APEX diffractometer
Absorption correction: multi-scan (*SADABS*; Sheldrick, 1996 ▶) *T*_min_ = 0.840, *T*_max_ = 0.966
17659 measured reflections
4445 independent reflections
3576 reflections with *I* > σ(*I*)
*R*_int_ = 0.037

#### Refinement

*R*[*F* ^2^ > 2σ(*F* ^2^)] = 0.046
*wR*(*F* ^2^) = 0.123
*S* = 1.05
4445 reflections
247 parameters
H-atom parameters constrained
Δρ_max_ = 0.39 e Å^−3^
Δρ_min_ = −0.36 e Å^−3^

### 

Data collection: *APEX2* (Bruker, 2008 ▶); cell refinement: *SAINT* (Bruker, 2008 ▶); data reduction: *SAINT*; program(s) used to solve structure: *SHELXS97* (Sheldrick, 2008 ▶); program(s) used to refine structure: *SHELXL97* (Sheldrick, 2008 ▶); molecular graphics: *X-SEED* (Barbour, 2001 ▶); software used to prepare material for publication: *publCIF* (Westrip, 2009 ▶).

## Supplementary Material

Crystal structure: contains datablocks global, I. DOI: 10.1107/S1600536809005923/sj2579sup1.cif

Structure factors: contains datablocks I. DOI: 10.1107/S1600536809005923/sj2579Isup2.hkl

Additional supplementary materials:  crystallographic information; 3D view; checkCIF report

## Figures and Tables

**Fig. 1.**
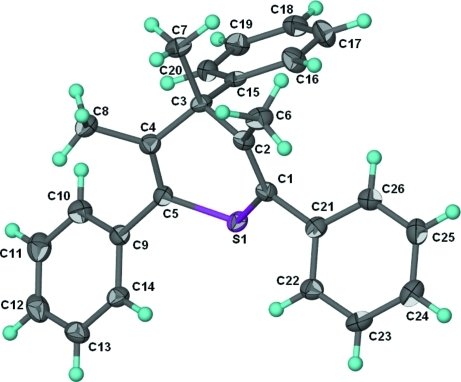
Thermal ellisoid plot (Barbour, 2001) of C26H24S; probability levels are set at 70% and H-atoms are drawn as spheres of arbitrary radius.

## supplementary crystallographic information

### Experimental

4-Methyl-2,4,6-triphenyl-4*H*-thiopyran was was synthesized by the reaction of methyl magnesium bromide and 3,5-dimethyl-2,4,6triphenyl thiopyrylium perchlorate in dry ether under an argon atmosphere according to a reported method (Rahmani & Pirelahi, 1997). The product was isolated by TLC on neutral alumina (petroleum ether 40–60 °C) and purified by recrystalization from ethanol.

### Refinement

Carbon-bound H-atoms were placed in calculated positions (C–H 0.95 to 0.98 Å) and were included in the refinement in the riding model approximation, with *U*(H) set to 1.2 to 1.5*U*(C).

### Crystal data

**Table:**

C_26_H_24_S	*F*(000) = 784
*M**_r_* = 368.51	*D*_x_ = 1.261 Mg m^−^^3^
Monoclinic, *P*2_1_/*n*	Mo *K*α radiation, λ = 0.71073 Å
Hall symbol: -P 2yn	Cell parameters from 5510 reflections
*a* = 8.4525 (1) Å	θ = 2.5–28.2°
*b* = 14.4732 (2) Å	µ = 0.17 mm^−^^1^
*c* = 16.2971 (3) Å	*T* = 115 K
β = 103.156 (1)°	Block, colorless
*V* = 1941.37 (5) Å^3^	0.35 × 0.35 × 0.20 mm
*Z* = 4	

### Data collection

**Table:**

Bruker SMART APEX diffractometer	4445 independent reflections
Radiation source: fine-focus sealed tube	3576 reflections with *I* > σ(*I*)
graphite	*R*_int_ = 0.037
ω scans	θ_max_ = 27.5°, θ_min_ = 1.9°
Absorption correction: multi-scan (*SADABS*; Sheldrick, 1996)	*h* = −10→10
*T*_min_ = 0.840, *T*_max_ = 0.966	*k* = −18→18
17659 measured reflections	*l* = −21→21

### Refinement

**Table:**

Refinement on *F*^2^	Primary atom site location: structure-invariant direct methods
Least-squares matrix: full	Secondary atom site location: difference Fourier map
*R*[*F*^2^ > 2σ(*F*^2^)] = 0.046	Hydrogen site location: inferred from neighbouring sites
*wR*(*F*^2^) = 0.123	H-atom parameters constrained
*S* = 1.05	*w* = 1/[σ^2^(*F*_o_^2^) + (0.059*P*)^2^ + 1.0605*P*] where *P* = (*F*_o_^2^ + 2*F*_c_^2^)/3
4445 reflections	(Δ/σ)_max_ = 0.001
247 parameters	Δρ_max_ = 0.39 e Å^−^^3^
0 restraints	Δρ_min_ = −0.36 e Å^−^^3^

### Fractional atomic coordinates and isotropic or equivalent isotropic displacement parameters (Å^2^)

**Table:**

	*x*	*y*	*z*	*U*_iso_*/*U*_eq_	
S1	0.66266 (5)	0.60193 (3)	0.30451 (3)	0.01781 (12)	
C1	0.56658 (19)	0.69496 (11)	0.34341 (10)	0.0168 (3)	
C2	0.4182 (2)	0.68372 (11)	0.35910 (10)	0.0170 (3)	
C3	0.32243 (19)	0.59530 (11)	0.32806 (11)	0.0165 (3)	
C4	0.4297 (2)	0.51230 (12)	0.36251 (10)	0.0174 (3)	
C5	0.5796 (2)	0.50682 (11)	0.34781 (10)	0.0166 (3)	
C6	0.3438 (2)	0.75486 (13)	0.40643 (12)	0.0231 (4)	
H6A	0.4224	0.8045	0.4257	0.035*	
H6B	0.3145	0.7257	0.4552	0.035*	
H6C	0.2462	0.7806	0.3692	0.035*	
C7	0.1580 (2)	0.59255 (13)	0.35402 (12)	0.0223 (4)	
H7A	0.1768	0.5943	0.4156	0.033*	
H7B	0.1002	0.5356	0.3329	0.033*	
H7C	0.0924	0.6460	0.3300	0.033*	
C8	0.3661 (2)	0.43945 (13)	0.41291 (11)	0.0224 (4)	
H8A	0.4540	0.3966	0.4374	0.034*	
H8B	0.2777	0.4053	0.3759	0.034*	
H8C	0.3253	0.4691	0.4581	0.034*	
C9	0.6879 (2)	0.42489 (11)	0.36408 (10)	0.0173 (3)	
C10	0.6295 (2)	0.33811 (12)	0.33442 (11)	0.0228 (4)	
H10	0.5200	0.3320	0.3038	0.027*	
C11	0.7283 (2)	0.26059 (13)	0.34882 (12)	0.0281 (4)	
H11	0.6859	0.2019	0.3289	0.034*	
C12	0.8894 (2)	0.26882 (13)	0.39231 (13)	0.0287 (4)	
H12	0.9577	0.2159	0.4021	0.034*	
C13	0.9494 (2)	0.35449 (14)	0.42122 (12)	0.0261 (4)	
H13	1.0595	0.3602	0.4510	0.031*	
C14	0.8508 (2)	0.43231 (13)	0.40722 (11)	0.0202 (4)	
H14	0.8941	0.4909	0.4270	0.024*	
C15	0.27841 (19)	0.59420 (11)	0.23067 (11)	0.0165 (3)	
C16	0.2352 (2)	0.67542 (12)	0.18567 (12)	0.0240 (4)	
H16	0.2420	0.7325	0.2151	0.029*	
C17	0.1825 (2)	0.67490 (13)	0.09868 (12)	0.0270 (4)	
H17	0.1534	0.7312	0.0692	0.032*	
C18	0.1722 (2)	0.59257 (12)	0.05465 (11)	0.0219 (4)	
H18	0.1364	0.5921	−0.0050	0.026*	
C19	0.2142 (2)	0.51143 (12)	0.09817 (11)	0.0219 (4)	
H19	0.2074	0.4546	0.0684	0.026*	
C20	0.2667 (2)	0.51212 (12)	0.18537 (11)	0.0201 (4)	
H20	0.2951	0.4555	0.2145	0.024*	
C21	0.6639 (2)	0.78164 (11)	0.35383 (10)	0.0178 (3)	
C22	0.8276 (2)	0.78257 (12)	0.39556 (11)	0.0201 (4)	
H22	0.8794	0.7268	0.4178	0.024*	
C23	0.9153 (2)	0.86471 (13)	0.40479 (12)	0.0237 (4)	
H23	1.0261	0.8647	0.4342	0.028*	
C24	0.8427 (2)	0.94655 (13)	0.37156 (12)	0.0251 (4)	
H24	0.9032	1.0025	0.3779	0.030*	
C25	0.6807 (2)	0.94582 (13)	0.32899 (11)	0.0240 (4)	
H25	0.6298	1.0016	0.3061	0.029*	
C26	0.5927 (2)	0.86423 (12)	0.31960 (11)	0.0207 (4)	
H26	0.4824	0.8645	0.2895	0.025*	

### Atomic displacement parameters (Å^2^)

**Table:**

	*U*^11^	*U*^22^	*U*^33^	*U*^12^	*U*^13^	*U*^23^
S1	0.0159 (2)	0.0162 (2)	0.0235 (2)	0.00008 (15)	0.00897 (16)	0.00014 (16)
C1	0.0155 (8)	0.0169 (8)	0.0175 (8)	0.0015 (6)	0.0030 (6)	−0.0009 (6)
C2	0.0155 (8)	0.0177 (8)	0.0174 (8)	0.0014 (6)	0.0034 (6)	−0.0006 (6)
C3	0.0118 (7)	0.0179 (8)	0.0207 (8)	−0.0009 (6)	0.0054 (6)	−0.0014 (6)
C4	0.0168 (8)	0.0188 (8)	0.0163 (8)	−0.0016 (6)	0.0030 (6)	−0.0007 (6)
C5	0.0166 (8)	0.0163 (8)	0.0170 (8)	−0.0015 (6)	0.0037 (6)	0.0009 (6)
C6	0.0193 (9)	0.0250 (9)	0.0262 (9)	−0.0004 (7)	0.0077 (7)	−0.0062 (7)
C7	0.0153 (8)	0.0278 (9)	0.0254 (9)	−0.0012 (7)	0.0082 (7)	−0.0013 (7)
C8	0.0193 (9)	0.0247 (9)	0.0244 (9)	−0.0028 (7)	0.0075 (7)	0.0031 (7)
C9	0.0164 (8)	0.0189 (8)	0.0177 (8)	0.0008 (6)	0.0062 (6)	0.0019 (6)
C10	0.0217 (9)	0.0229 (9)	0.0237 (9)	−0.0003 (7)	0.0050 (7)	−0.0012 (7)
C11	0.0338 (11)	0.0217 (9)	0.0317 (10)	0.0018 (8)	0.0133 (9)	0.0009 (8)
C12	0.0294 (10)	0.0257 (10)	0.0351 (10)	0.0113 (8)	0.0160 (8)	0.0091 (8)
C13	0.0177 (9)	0.0349 (11)	0.0267 (9)	0.0053 (8)	0.0069 (7)	0.0094 (8)
C14	0.0161 (8)	0.0244 (9)	0.0210 (8)	−0.0016 (7)	0.0058 (7)	0.0023 (7)
C15	0.0092 (7)	0.0202 (8)	0.0207 (8)	−0.0001 (6)	0.0044 (6)	0.0002 (6)
C16	0.0274 (9)	0.0162 (8)	0.0263 (9)	0.0046 (7)	0.0016 (7)	−0.0038 (7)
C17	0.0314 (10)	0.0194 (9)	0.0271 (10)	0.0042 (7)	0.0000 (8)	0.0032 (7)
C18	0.0181 (8)	0.0265 (9)	0.0200 (8)	0.0004 (7)	0.0019 (7)	−0.0008 (7)
C19	0.0207 (8)	0.0186 (8)	0.0251 (9)	−0.0001 (7)	0.0027 (7)	−0.0044 (7)
C20	0.0188 (8)	0.0167 (8)	0.0239 (9)	−0.0007 (6)	0.0030 (7)	0.0014 (7)
C21	0.0177 (8)	0.0193 (8)	0.0177 (8)	−0.0010 (6)	0.0067 (6)	−0.0012 (6)
C22	0.0172 (8)	0.0211 (9)	0.0221 (8)	0.0012 (6)	0.0050 (7)	0.0000 (7)
C23	0.0160 (8)	0.0286 (10)	0.0267 (9)	−0.0041 (7)	0.0050 (7)	−0.0019 (7)
C24	0.0271 (10)	0.0227 (9)	0.0277 (10)	−0.0061 (7)	0.0106 (8)	−0.0022 (7)
C25	0.0274 (10)	0.0195 (9)	0.0256 (9)	−0.0002 (7)	0.0070 (8)	0.0038 (7)
C26	0.0188 (8)	0.0223 (9)	0.0207 (8)	0.0004 (7)	0.0039 (7)	−0.0005 (7)

### Geometric parameters (Å, °)

**Table:**

S1—C1	1.7631 (17)	C12—C13	1.381 (3)
S1—C5	1.7638 (16)	C12—H12	0.9500
C1—C2	1.346 (2)	C13—C14	1.389 (3)
C1—C21	1.488 (2)	C13—H13	0.9500
C2—C6	1.507 (2)	C14—H14	0.9500
C2—C3	1.537 (2)	C15—C16	1.390 (2)
C3—C4	1.532 (2)	C15—C20	1.390 (2)
C3—C7	1.543 (2)	C16—C17	1.386 (3)
C3—C15	1.546 (2)	C16—H16	0.9500
C4—C5	1.345 (2)	C17—C18	1.383 (2)
C4—C8	1.509 (2)	C17—H17	0.9500
C5—C9	1.485 (2)	C18—C19	1.376 (2)
C6—H6A	0.9800	C18—H18	0.9500
C6—H6B	0.9800	C19—C20	1.389 (2)
C6—H6C	0.9800	C19—H19	0.9500
C7—H7A	0.9800	C20—H20	0.9500
C7—H7B	0.9800	C21—C26	1.396 (2)
C7—H7C	0.9800	C21—C22	1.397 (2)
C8—H8A	0.9800	C22—C23	1.391 (2)
C8—H8B	0.9800	C22—H22	0.9500
C8—H8C	0.9800	C23—C24	1.386 (3)
C9—C10	1.395 (2)	C23—H23	0.9500
C9—C14	1.400 (2)	C24—C25	1.387 (3)
C10—C11	1.387 (3)	C24—H24	0.9500
C10—H10	0.9500	C25—C26	1.386 (2)
C11—C12	1.389 (3)	C25—H25	0.9500
C11—H11	0.9500	C26—H26	0.9500
			
C1—S1—C5	101.20 (8)	C13—C12—C11	119.49 (17)
C2—C1—C21	126.50 (15)	C13—C12—H12	120.3
C2—C1—S1	120.10 (13)	C11—C12—H12	120.3
C21—C1—S1	113.39 (12)	C12—C13—C14	120.77 (17)
C1—C2—C6	122.02 (15)	C12—C13—H13	119.6
C1—C2—C3	118.81 (14)	C14—C13—H13	119.6
C6—C2—C3	119.17 (14)	C13—C14—C9	120.34 (17)
C4—C3—C2	107.98 (13)	C13—C14—H14	119.8
C4—C3—C7	111.96 (14)	C9—C14—H14	119.8
C2—C3—C7	111.78 (13)	C16—C15—C20	117.66 (16)
C4—C3—C15	110.63 (13)	C16—C15—C3	120.41 (15)
C2—C3—C15	109.40 (13)	C20—C15—C3	121.70 (15)
C7—C3—C15	105.07 (13)	C17—C16—C15	121.32 (16)
C5—C4—C8	121.52 (15)	C17—C16—H16	119.3
C5—C4—C3	118.93 (14)	C15—C16—H16	119.3
C8—C4—C3	119.54 (14)	C18—C17—C16	120.20 (17)
C4—C5—C9	125.67 (15)	C18—C17—H17	119.9
C4—C5—S1	120.16 (13)	C16—C17—H17	119.9
C9—C5—S1	114.17 (12)	C19—C18—C17	119.29 (17)
C2—C6—H6A	109.5	C19—C18—H18	120.4
C2—C6—H6B	109.5	C17—C18—H18	120.4
H6A—C6—H6B	109.5	C18—C19—C20	120.40 (16)
C2—C6—H6C	109.5	C18—C19—H19	119.8
H6A—C6—H6C	109.5	C20—C19—H19	119.8
H6B—C6—H6C	109.5	C19—C20—C15	121.13 (16)
C3—C7—H7A	109.5	C19—C20—H20	119.4
C3—C7—H7B	109.5	C15—C20—H20	119.4
H7A—C7—H7B	109.5	C26—C21—C22	118.38 (16)
C3—C7—H7C	109.5	C26—C21—C1	119.97 (15)
H7A—C7—H7C	109.5	C22—C21—C1	121.63 (15)
H7B—C7—H7C	109.5	C23—C22—C21	120.38 (16)
C4—C8—H8A	109.5	C23—C22—H22	119.8
C4—C8—H8B	109.5	C21—C22—H22	119.8
H8A—C8—H8B	109.5	C24—C23—C22	120.70 (17)
C4—C8—H8C	109.5	C24—C23—H23	119.6
H8A—C8—H8C	109.5	C22—C23—H23	119.6
H8B—C8—H8C	109.5	C23—C24—C25	119.19 (17)
C10—C9—C14	118.23 (16)	C23—C24—H24	120.4
C10—C9—C5	120.13 (15)	C25—C24—H24	120.4
C14—C9—C5	121.62 (15)	C26—C25—C24	120.39 (17)
C11—C10—C9	121.18 (17)	C26—C25—H25	119.8
C11—C10—H10	119.4	C24—C25—H25	119.8
C9—C10—H10	119.4	C25—C26—C21	120.93 (16)
C10—C11—C12	119.97 (18)	C25—C26—H26	119.5
C10—C11—H11	120.0	C21—C26—H26	119.5
C12—C11—H11	120.0		
			
C5—S1—C1—C2	−29.19 (15)	C10—C11—C12—C13	0.3 (3)
C5—S1—C1—C21	151.99 (12)	C11—C12—C13—C14	−0.1 (3)
C21—C1—C2—C6	−12.4 (3)	C12—C13—C14—C9	0.6 (3)
S1—C1—C2—C6	168.91 (13)	C10—C9—C14—C13	−1.3 (2)
C21—C1—C2—C3	167.85 (15)	C5—C9—C14—C13	−179.74 (16)
S1—C1—C2—C3	−10.8 (2)	C4—C3—C15—C16	−156.56 (15)
C1—C2—C3—C4	54.54 (19)	C2—C3—C15—C16	−37.7 (2)
C6—C2—C3—C4	−125.18 (16)	C7—C3—C15—C16	82.42 (18)
C1—C2—C3—C7	178.12 (15)	C4—C3—C15—C20	29.1 (2)
C6—C2—C3—C7	−1.6 (2)	C2—C3—C15—C20	147.93 (15)
C1—C2—C3—C15	−65.93 (19)	C7—C3—C15—C20	−91.92 (18)
C6—C2—C3—C15	114.35 (16)	C20—C15—C16—C17	−0.1 (3)
C2—C3—C4—C5	−54.04 (19)	C3—C15—C16—C17	−174.63 (16)
C7—C3—C4—C5	−177.52 (15)	C15—C16—C17—C18	−0.1 (3)
C15—C3—C4—C5	65.65 (19)	C16—C17—C18—C19	0.2 (3)
C2—C3—C4—C8	125.17 (16)	C17—C18—C19—C20	−0.1 (3)
C7—C3—C4—C8	1.7 (2)	C18—C19—C20—C15	−0.1 (3)
C15—C3—C4—C8	−115.13 (16)	C16—C15—C20—C19	0.2 (3)
C8—C4—C5—C9	11.4 (3)	C3—C15—C20—C19	174.69 (15)
C3—C4—C5—C9	−169.35 (15)	C2—C1—C21—C26	−49.4 (2)
C8—C4—C5—S1	−169.24 (13)	S1—C1—C21—C26	129.31 (14)
C3—C4—C5—S1	10.0 (2)	C2—C1—C21—C22	132.24 (18)
C1—S1—C5—C4	29.71 (15)	S1—C1—C21—C22	−49.03 (19)
C1—S1—C5—C9	−150.90 (12)	C26—C21—C22—C23	2.0 (2)
C4—C5—C9—C10	50.0 (2)	C1—C21—C22—C23	−179.63 (16)
S1—C5—C9—C10	−129.34 (15)	C21—C22—C23—C24	−1.1 (3)
C4—C5—C9—C14	−131.58 (18)	C22—C23—C24—C25	0.2 (3)
S1—C5—C9—C14	49.07 (19)	C23—C24—C25—C26	−0.2 (3)
C14—C9—C10—C11	1.5 (3)	C24—C25—C26—C21	1.1 (3)
C5—C9—C10—C11	179.99 (16)	C22—C21—C26—C25	−2.0 (3)
C9—C10—C11—C12	−1.0 (3)	C1—C21—C26—C25	179.61 (16)
